# Supplementary material for: Impact of hospital accreditation on quality improvement in healthcare: A systematic review
Source: PLoS One. 2023 Dec 5;18(12):e0294180. doi: 10.1371/journal.pone.0294180 (PMC10697559; doi:10.1371/journal.pone.0294180)
Supplement: S2 File — (DOCX) [file pone.0294180.s002.docx]

**S2:** **A complete description of the result of electronic** **search strategies of two databases (Ovid MEDLINE and PUBMED)**

**Database(s):**Ovid MEDLINE(R) and Epub Ahead of Print, In-Process & Other Non-Indexed Citations, Daily and Versions(R) 1946 to December 31, 2021

**Search date:** January 17, 2022

**Search Strategy:**

| **#** | **Searches** |
| --- | --- |
| 1 | exp Accreditation/ |
| 2 | accredit*.mp. [mp=title, abstract, original title, name of substance word, subject heading word, floating sub-heading word, keyword heading word, organism supplementary concept word, protocol supplementary concept word, rare disease supplementary concept word, unique identifier, synonyms] |
| 3 | 1 or 2 |
| 4 | exp Hospitals/ |
| 5 | exp "Quality of Health Care"/ or exp Total Quality Management/ or exp Quality Improvement/ or exp Quality Control/ or exp "Quality of Life"/ or exp Quality Assurance, Health Care/ or exp Quality Indicators, Health Care/ |
| 6 | 3 and 4 and 5 |
| 7 | limit 6 to (english language and english and last 10 years) |
| 8 | hospital.mp. or exp Hospitals/ |
| 9 | exp "Joint Commission on Accreditation of Healthcare Organizations"/ or exp Accreditation/ or Accreditation.mp. |
| 10 | certification.mp. or exp Certification/ |
| 11 | exp Licensure, Hospital/ |
| 12 | accredit*.mp. |
| 13 | 9 or 10 or 11 or 12 |
| 14 | exp Quality Assurance, Health Care/ or exp "Joint Commission on Accreditation of Healthcare Organizations"/ |
| 15 | exp Quality Improvement/ or exp Total Quality Management/ or exp "Quality of Health Care"/ |
| 16 | 14 or 15 |
| 17 | "Outcome Assessment (Health Care)"/ |
| 18 | exp "Process Assessment (Health Care)"/ |
| 19 | 17 or 18 |
| 20 | 8 and 13 and 16 and 19 |
| 21 | limit 20 to (English language and yr="2009 - 2019") |

**Database:** PUBMED

**Search date:** January 17, 2022

**Search Strategy:**

| Search | Query |
| --- | --- |
| #11 | Search ((((((accreditation) AND quality improvement)) AND (hospital[tiab] AND accredit*[tiab] AND quality[tiab])) AND (((((hospital) AND (accredit* OR certification OR licensure)) AND (quality improvement OR quality assurance OR total quality management)) AND (outcome OR process assessment)) AND (patient satisfaction OR patient safety))) AND (((((hospital) AND (accredit* OR certification OR licensure)) AND (quality improvement OR quality assurance OR total quality management)) AND (outcome OR process assessment)) AND (patient satisfaction OR patient safety))) AND hospital[tiab] AND (("2009/01/01"[PDat] : "2019/12/31"[PDat])) |
| [#10](https://www.ncbi.nlm.nih.gov/pubmed/advanced) | Search ((((((accreditation) AND quality improvement)) AND (hospital[tiab] AND accredit*[tiab] AND quality[tiab])) AND (((((hospital) AND (accredit* OR certification OR licensure)) AND (quality improvement OR quality assurance OR total quality management)) AND (outcome OR process assessment)) AND (patient satisfaction OR patient safety))) AND (((((hospital) AND (accredit* OR certification OR licensure)) AND (quality improvement OR quality assurance OR total quality management)) AND (outcome OR process assessment)) AND (patient satisfaction OR patient safety))) AND hospital[tiab] Filters: Publication date from 2009/01/01 to 2019/12/31 |
| #9 | Search ((((((accreditation) AND quality improvement)) AND (hospital[tiab] AND accredit*[tiab] AND quality[tiab])) AND (((((hospital) AND (accredit* OR certification OR licensure)) AND (quality improvement OR quality assurance OR total quality management)) AND (outcome OR process assessment)) AND (patient satisfaction OR patient safety))) AND (((((hospital) AND (accredit* OR certification OR licensure)) AND (quality improvement OR quality assurance OR total quality management)) AND (outcome OR process assessment)) AND (patient satisfaction OR patient safety))) AND hospital[tiab] |
| #8 | Search ((((((accreditation) AND quality improvement)) AND (hospital[tiab] AND accredit*[tiab] AND quality[tiab])) AND (((((hospital) AND (accredit* OR certification OR licensure)) AND (quality improvement OR quality assurance OR total quality management)) AND (outcome OR process assessment)) AND (patient satisfaction OR patient safety))) AND (((((hospital) AND (accredit* OR certification OR licensure)) AND (quality improvement OR quality assurance OR total quality management)) AND (outcome OR process assessment)) AND (patient satisfaction OR patient safety))) AND hospital[tiab] Filters: published in the last 10 years |
| #7 | Search ((((((accreditation) AND quality improvement)) AND (hospital [tiab] AND accredit*[tiab] AND quality [tiab])) AND (((((hospital) AND (accredit* or certification or licensure)) AND (quality improvement or quality assurance or total quality management)) AND (outcome or process assessment)) AND (patient staisfaction or patient safety))) AND (((((hospital) AND (accredit* OR certification OR licensure)) AND (quality improvement OR quality assurance OR total quality management)) AND (outcome OR process assessment)) AND (patient satisfaction OR patient safety))) AND hospital [tiab] |
| #6 | Search hospital [tiab] |
| #5 | Search ((((hospital) AND (accredit* OR certification OR licensure)) AND (quality improvement OR quality assurance OR total quality management)) AND (outcome OR process assessment)) AND (patient satisfaction OR patient safety) |
| #4 | Search ((((hospital) AND (accredit* or certification or licensure)) AND (quality improvement or quality assurance or total quality management)) AND (outcome or process assessment)) AND (patient staisfaction or patient safety) |
| #3 | Search hospital [tiab] AND accredit*[tiab] AND quality [tiab] |
| #1 | Search (accreditation) AND quality improvement |
