# Supplementary material for: Impact of hospital accreditation on quality improvement in healthcare: A systematic review
Source: PLoS One. 2023 Dec 5;18(12):e0294180. doi: 10.1371/journal.pone.0294180 (PMC10697559; doi:10.1371/journal.pone.0294180)
Supplement: S3 File — (DOCX) [file pone.0294180.s003.docx]

**S3:** **Data extraction sheet for potentially relevant studies on full-text screening level**

| **Study Reference ID** (first author, year)**:** |
| --- |
| **Study Title:** |
| **Study Authors:** |
| **Publication Information:**  Year: __________ Journal name: ____________  Volume: __________ Issue: _________ Page numbers________−________ |
| **Study Citation:** |
| **Study Setting (country; city):** |
| **Duration of the study/ Period of data collection:** |
| **Aims of the study:** |
| **Decision to include or exclude the study, + reason if excluded:**   - No eligible population. - No eligible intervention. - Inadequate outcome. - Letter/opinion/editorial/ commentary/ conference reports, or abstract format. - Ineligible study design |
| **Study Design** (e.g., qualitative, mixed methods, intervention study): |
| **Study data:**   - Total number of hospital/ healthcare professional participants (e.g. four hospitals, 400 healthcare professionals). - Type of organization participant (e.g. public, private, or teaching hospital). - Type of healthcare professional participant in the study (e.g. healthcare managers, nurses, physicians, allied healthcare professionals). - Participant's characteristics (age/sex): - Methodological/statistical approach to identifying accreditation impact and/or contextual factors of accreditation implementation. |
| **Study methods:**   - methods used to study accreditation impact and/or contextual factors of accreditation implementation (e.g. interview questions, surveys). - Data analysis methods (e.g. statistical or quantitative method) |
| **Study Outcomes:**   - Quantitative results - Qualitative results/ contextual factors influencing implementation of accreditation programs |
| **Study implications** (e.g. recommendations for healthcare organizations made based on the findings): |
| **Study Limitations:** |
| **Informed consent obtained:**  () Yes () No () Unclear |
| **Study Funding Source:** |
| **Possible conflicts of interest:**(for study authors) |
