# Supplementary material for: Impact of hospital accreditation on quality improvement in healthcare: A systematic review
Source: PLoS One. 2023 Dec 5;18(12):e0294180. doi: 10.1371/journal.pone.0294180 (PMC10697559; doi:10.1371/journal.pone.0294180)
Supplement: S4 File — (DOCX) [file pone.0294180.s004.docx]

**S4: Normalization Process Theory (NPT) Coding Framework Used for Analysis of Review Data on Hospital Accreditation Implementation** (May et al., 2011; May et al., 2015)

| **Construct** | **Sub construct** | **Description** | **Code** |
| --- | --- | --- | --- |
| **Coherence**  **(Sense-making work)** | **Differentiation** | Is there a clear understanding of how accreditation process as a new way of working differs from existing practice? | **CODI** |
|  | **Communal specification** | Do individuals have a shared understanding of the aims, objectives and potential benefits of the accreditation program? | **COCS** |
|  | **Individual specification** | Do individuals have a clear understanding of their specific tasks and responsibilities in the implementation of accreditation program? Does it make sense to them? | **COIS** |
|  | **Internalization** | Do individuals understand the value, benefits and importance of the accreditation program? | **COIN** |
| **Cognitive participation (Relationship work)** | **Enrolment** | Do individuals “buy into” the idea of the accreditation program? Will they invest time and effort on the implementation process? | **CPEN** |
|  | **Activation** | Can individuals sustain involvement? Do they think they have required support from accreditation specialist and key persons to take forward the implementation process? | **CPAC** |
|  | **Initiation** | Are key individuals willing to drive the implementation? Can they engage others in the implementation process? | **CPIN** |
|  | **Legitimation** | Do individuals believe it is right for them to be involved in the accreditation program? Are they seeking reassurance from others about the appropriateness of the implementation plan? | **CPLE** |
| **Collective action (Enacting or operational work)** | **Skill set workability** | How does the accreditation process affect roles and responsibilities or training needs of those implementing the accreditation? | **CASW** |
|  | **Contextual Integration** | Is there organizational support to enable the implementation of accreditation program? (e.g., financial, human, or social resources support) | **CACI** |
|  | **Interactional workability** | Does the accreditation program make people’s work easier or harder? | **CAIW** |
|  | **Relational integration** | Do individuals have confidence in the accreditation process and in others implementing it? | **CARI** |
| **Reflexive monitoring (Appraisal work)** | **Reconfiguration** | Do individuals try to alter or redefine the accreditation process based on evaluation and experience? | **RMRE** |
|  | **Communal appraisal** | How do groups judge the value and effectiveness of the accreditation program? | **RMCA** |
|  | **Individual appraisal** | How do individuals appraise the effects of the accreditation process on them and their work environment? | **RMIA** |
|  | **Systematization** | How are benefits or problems identified or measured by individuals? How they are evaluating the impact and benefits of the accreditation? This may use formal and/or informal methods. | **RMSY** |
