# Supplementary material for: Impact of hospital accreditation on quality improvement in healthcare: A systematic review
Source: PLoS One. 2023 Dec 5;18(12):e0294180. doi: 10.1371/journal.pone.0294180 (PMC10697559; doi:10.1371/journal.pone.0294180)
Supplement: S6 File — (DOCX) [file pone.0294180.s006.docx]

**S6: The quality assessment tool for included articles drawn from Hawker et al., 2002**

Please assess each paper on the following criteria. For scoring, please refer to notes below.

Good=4

Fair=3

Poor=2

Very poor=1

Lower scores =poor quality

**Notes for appraising the quality of each paper:**

| **1. Abstract and title:**  Did they provide a clear description of the study?  Good Structured abstract with full information and clear title.  Fair Abstract with most of the information.  Poor Inadequate abstract.  Very Poor No abstract.  **2. Introduction and aims:**  Was there a good background and clear statement of the aims of the research?  Good Full but concise background to discussion/study containing up-to date literature review and highlighting gaps in knowledge. Clear statement of aim AND objectives including research questions.  Fair Some background and literature review. Research questions outlined.  Poor Some background but no aim/objectives/questions, OR Aims/objectives but inadequate background.  Very Poor No mention of aims/objectives. No background or literature review.  **3. Method and data:**  Is the method appropriate and clearly explained?  Good Method is appropriate and described clearly (e.g., questionnaires included). Clear details of the data collection and recording.  Fair Method appropriate, description could be better. Data described.  Poor Questionable whether method is appropriate. Method described inadequately. Little description of data.  Very Poor No mention of method, AND/OR Method inappropriate, AND/OR No details of data.    **4.** **Sampling:**  Was the sampling strategy appropriate to address the aims?  Good Details (age/gender/race/context) of who was studied and how they were recruited. Why this group was targeted. The sample size was justified for the study. Response rates shown and explained.  Fair Sample size justified. Most information given, but some missing.  Poor Sampling mentioned but few descriptive details.  Very Poor No details of sample.    5. **Data analysis:**  Was the description of the data analysis sufficiently rigorous?  Good Clear description of how analysis was done. Qualitative studies: Description of how themes derived/ respondent validation or triangulation. Quantitative studies: Reasons for tests selected hypothesis driven/ numbers add up/statistical significance discussed.  Fair Qualitative: Descriptive discussion of analysis. Quantitative.  Poor Minimal details about analysis.  Very Poor No discussion of analysis.  6. **Ethics and bias:**  Have ethical issues been addressed, and what has necessary ethical approval gained? Has the relationship between researchers and participants been adequately considered?  Good Ethics: Where necessary issues of confidentiality, sensitivity, and consent were addressed. Bias: Researcher was reflexive and/or aware of own bias.  Fair Lip service was paid to above (i.e., these issues were acknowledged).  Poor Brief mention of issues.  Very Poor No mention of issues.    **7. Results:**  Is there a clear statement of the findings?  Good Findings explicit, easy to understand, and in logical progression. Tables, if present, are explained in text. Results relate directly to aims. Sufficient data are presented to support findings.  Fair Findings mentioned but more explanation could be given. Data presented relate directly to results.  Poor Findings presented haphazardly, not explained, and do not progress logically from results.  Very Poor Findings not mentioned or do not relate to aims.    8. **Transferability or generalizability:**  Are the findings of this study transferable (generalizable) to a wider population?  Good Context and setting of the study are described sufficiently to allow comparison with other contexts and settings, plus high score in Question 4 (sampling).  Fair Some context and setting described, but more needed to replicate or compare the study with others, PLUS fair score or higher in Question 4.  Poor Minimal description of context/setting.  Very Poor No description of context/setting.    9. **Implications and usefulness: How important are these findings to policy and practice?**  Good Contributes something new and/or different in terms of understanding/insight or perspective. Suggests ideas for further research. Suggests implications for policy and/or practice.  Fair Two of the above (state what is missing in comments).  Poor Only one of the above.  Very Poor None of the above. |
| --- |
