# Supplementary material for: Impact of hospital accreditation on quality improvement in healthcare: A systematic review
Source: PLoS One. 2023 Dec 5;18(12):e0294180. doi: 10.1371/journal.pone.0294180 (PMC10697559; doi:10.1371/journal.pone.0294180)
Supplement: S7 File — (DOCX) [file pone.0294180.s007.docx]

**S7 File: The included and excluded studies and reasons for exclusion**

**Full-Text articles included in the final review**

1. Ajarmah, B.S. & Hashem, T.N. (2015) Patient satisfaction evaluation on hospitals; comparison study between accredited and non-accredited hospitals In Jordan. *European Scientific Journal*;11(32):298-314.
2. Almasabi, M., & Thomas, S. (2017) The impact of Saudi hospital accreditation on quality of care. *International Journal of Health Planning and Management*, 32(4), e261–e278
3. Bogh, S. B., Falstie-Jensen, A. M., Hollnagel, E., Holst, R., Braithwaite, J., & Johnsen, S. P. (2016) Improvement in quality of hospital care during accreditation a nationwide stepped wedge study. *International Journal for Quality in Health Care*, 28(6), 715–720. doi: 10.1093/intqhc/mzw099
4. Davis, M. V., Cannon, M. M., Corso, L., Lenaway, D., & Baker, E. L. (2009). Incentives to encourage participation in the national public health accreditation model: a systematic investigation. *American journal of public health*, 99(9), 1705–1711. <https://doi.org/10.2105/AJPH.2008.151118>
5. Desveaux, L., Mitchell, J., Shaw, J. & Ivers, N.M. (2017) Understanding the impact of accreditation on quality of healthcare: A grounded theory approach (NPT). *International Journal for Quality in Health Care*, 29(7), 941–947, doi: 10.1093/intqhc/mzx136
6. Devkaran, S. & O’Farrell, P. (2015) The impact of hospital accreditation on quality measures an interrupted time series analysis. *BMC Health Services Research*, 15, 137. DOI 10.1186/s12913-015-0784-5
7. Ehlers, L., Jensen, M., Simonsen, K., Rasmussen, G. & Braithwaite, J. (2017) Attitudes towards accreditation among hospital employees in Denmark: a cross-sectional survey. *International Journal for Quality in Health Care*, 29(5), 693-698. doi: 10.1093/intqhc/mzx090.
8. Halasa, Y.A., Zeng, W., Chappy, E. & Shepard, D.S. (2015) Value and impact of international hospital accreditation: a case study from Jordan. *East Mediterranean Health Journal,* 21(2), 90-99.
9. Hijazi, H., Harvey, H., Alyahya, M., Alshraideh, H., Al Abdi, R. & Parahoo, S. (2018). The impact of applying QM practices on Patient Centeredness in Jordanian Public Hospitals: Results of Predictive Modeling. Inquiry: *The Journal of Health Care Organization, Provision, and Financing*, 55, 1–15
10. Hinchcliff, R., Greenfield, D., Hogden, A., Sarrami-Foroushani, P., Travaglia, J. & Braithwaite, J. (2016) Levers for change: an investigation of how accreditation programmes can promote consumer engagement in healthcare. *International Journal for Quality in Health Care,* 28 (5), 561–565.
11. Hinchcliff, R., Greenfield, D., Westbrook, J., Pawsey, M., Mumford, V. & Braithwaite, J. (2013) Stakeholder perspectives on implementing accreditation programs: a qualitative study of enabling factors. *BMC Health Services Research,* 13(1), 437. doi: 10.1186/1472-6963- 13-437.
12. Lutfiyya, N., Sikka, A., Mehta, S. & Lipsky, M. (2009) Comparison of US accredited and non-accredited rural critical access hospitals. *International Journal for Quality in Health Car*e, 21(2), 112-118.
13. Melo, S. (2016) The impact of accreditation on healthcare quality improvement: a qualitative case study. *Journal of Health Organization and Management*, 30(8), 1242-1258
14. Mumford et al (2015) Counting the costs of accreditation in acute care: an activity-based costing approach. BMJ Open; 5, e008850. doi:10.1136/bmjopen-2015-008850
15. Pomey, M., Lemieux-Charles, L., Champagne, F., Angus, D., Shabah, A. & Contandriopoulos, A. (2010) Does accreditation stimulate change? A study of the impact of the accreditation process on Canadian healthcare organizations. Implementation Science, 5, 1-14, doi: 10.1186/1748-5908-5-31.
16. Reisi, N, Raeissi, P, Sokhanvar, M. & Kakemam, E. (2019) The impact of accreditation on nurses' perceptions of quality of care in Iran and its barriers and facilitators. International Journal of Health Planning and Management; 34: e230– e240. https://doi.org/10.1002/hpm.2642
17. Saadati, M., Yarifard, K., Azami‐Agdash, S. & Tabrizi, J. S. (2015) Challenges and potential drivers of accreditation in the Iranian hospitals. *International Journal of Hospital Research*, 4(1), 37‐42.
18. Saif, NI. (2016) Quality of health services and patients’ satisfaction in accredited and non-accredited hospitals. International Journal of Business and Management, 11(10), 298-305.
19. Saleh, S.S., Bou Sleiman, J., Dagher, D., Sbeit, H. & Natafgi, N. (2013) Accreditation of hospitals in Lebanon: is it a worthy investment? International Journal for Quality in Health Care, 25(3), 1-7.
20. Yildiz & Kaya (2014) Perceptions of nurses on the impact of accreditation on Quality of care: A survey in a hospital in Turkey. Clinical Governance: An International Journal, 19(2), 69-82
21. Zarifraftar, M. (2018) Ranking of Challenges of Accreditation Standards Implementation in Public and private Hospitals: A comparative approach. UCT Journal of Management and Accounting Studies, 6(02), 45-54 DOI: https://doi.org/10.24200/

**Full-text articles excluded and reasons for exclusion (n = 131)**

1. **No eligible population: (n = 23)**

Non-hospital setting (e.g., primary healthcare organizations or other community-based healthcare organizations)

1. **No eligible intervention: (n = 46)**

Not general accreditation and/or certification programmes of hospitals but focus on single-specialty accreditation programmes (for example: oncology centre, radiology, surgical program, and medical education accreditation programmes)

1. **Inadequate outcome: (n=43)**

Not discussed the influence of hospital accreditation programmes on quality improvement of healthcare services, or identified the contextual factors influencing the effective implementation of hospital accreditation, or reported improvement of patient’s satisfaction and patient safety, or article evaluated the cost and financial impact of hospital accreditation

1. **Letter/opinion/editorial/ conference reports/** **Commentary/** **abstract format (complete study not available) (n = 14)**
2. **Ineligible study design: (n=5)**

**Characteristics of excluded studies [ordered by study ID]**

| **NO** | **Study Reference** | **Reason for Exclusion** |
| --- | --- | --- |
| **No eligible population: (n = 23)** | | |
| 1 | El-Jardali F, Hemadeh R, Jaafar M, et al. (2014) The impact of accreditation of primary healthcare centers: successes, challenges and policy implications as perceived by healthcare providersnand directors in Lebanon. BMC Health Serv Res., 14, 86.  doi:10.1186/1472-6963-14-86 | No eligible population: Primary healthcare centers not hospital |
| 2 | O'Beirne et al. (2013).The status of accreditation in primary care. Quality in Primary Care, 21, 23–31. | No eligible population: Primary healthcare centers not hospital |
| 3 | Gadallah et al. (2010). Are patients and healthcare providers satisfied with health sector Reform implemented in family health centres? Qual Saf Healthcare, 19, 1-5. | No eligible population: Primary healthcare centers not hospital |
| 4 | Campbell, S. M., Chauhan, U., & Lester, H. (2010). Primary medical care provider accreditation (PMCPA): pilot evaluation. British Journal General Practice, 60(576), e295-e304. | No eligible population: Primary healthcare centers not hospital |
| 5 | Murphy et al. (2013) Three Decades of Quality Improvement in Electroconvulsive Therapy: Exploring the Role of Accreditation. The Journal of ECT. 29(4):312–317  doi: 10.1097/YCT.0b013e3182926fc4 | No eligible population:  Electroconvulsive therapy clinics not hospital |
| 6 | van Doorn-Klomberg, A. L., Braspenning, J. C., Wolters, R. J., Bouma, M., & Wensing, M.  (2014). Effect of accreditation on the quality of chronic disease management: a  comparative observational study. BMC family practice, 15(1), 179. | No eligible population: Primary healthcare centers not hospital |
| 7 | Gareeb et al 2018 Examining the Impact of Accreditation on a primary healthcare organization in Qatar. BMC Medical Education; 18:216  https://doi.org/10.1186/s12909-018-1321-0 | No eligible population: Primary healthcare organization |
| 8 | El‐Jardali F, Ammar W, Hemadeh R, Jamal D, Jaafar M. 2013 Improving primary healthcare through accreditation: baseline assessment of readiness and challenges in lebanese context. Int J Health Plann Manage.;28(4):256‐279. | No eligible population: Primary healthcare setting |
| 9 | Hogden, A, Greenfield, D, Brandon, M, Debono, D, Mumford, V, Westbrook, J & Braithwaite, J 2017, 'How does accreditation influence staff perceptions of quality in residential aged care?', Quality in Ageing and Older Adults, vol. 18, no. 2, pp. 131-144. https://doi.org/10.1108/QAOA-07-2016-0028 | No eligible population: residential aged care centers |
| 10 | Al Tehewy N, Salem B, Habil I, El Okda S. Evaluation of accreditation program in non-governmental organisations' health units in Egypt: short term outcomes. International Journal for Quality in Healthcare 2009;21(3):183-189. | No eligible population:  non-governmental healthcare units- not hospital setting |
| 11 | AbouElnour, A., Hernan L.A., Ford, D., Clark, S., Fuller, D., Johnson, K.J., Dunbar, J.A. (2014). Surveyors' perceptions of the impact of accreditation on patient safety in general practice. Medical Journal of Australia, 201(3), 56-59. | No eligible population: General practice centers |
| 12 | El Jardali, F., Ammar, W., Hemadeh, R., Jamal, D., Jaafar, M. (2013). Improving primary healthcare through accreditation: baselines assessment of readiness and challenges in Lebanese context. The International Journal of Health Planning and Management, 28(4), 256-279. | No eligible population: primary healthcare centers |
| 13 | Macinko, J., & Starfield, B. (2009). The impact of primary health care on population health in low- and middle-income countries. Journal of Ambulatory Care Management, 32(2), 150–171. | No eligible population: primary healthcare centers |
| 14 | Nouwens, E., van Lieshout, J., & Wensing, M. (2015). Determinants of impact of a practice accreditation program in primary care: a qualitative study. BMC Family Practice, 16(1), 78. | No eligible population: primary healthcare centers |
| 15 | Waldorff, F. B., Nicolaisdottir, D. R., Kousgaard, M. B., Reventlow, S., Søndergaard, J., Thorsen, T., Andersen, M. K., Pedersen, L. B., Bisgaard, L., Hutters, C. L., Bro, F. (2016). Almost half of the Danish general practitioners have negative a priori attitudes towards a mandatory accreditation programme. Danish Medical Journal, 63(9), 1-5. | No eligible population:  General Practice centers |
| 16 | Alsakkak, M. A., Alwahabi, S. A., Alsalhi, H. M., & Shugdar, M. A. (2017). Outcome of the first Saudi Central Board for Accreditation of Healthcare Institutions (CBAHI) primary health care accreditation cycle in Saudi Arabia. Saudi medical journal, 38(11), 1132–1136. https://doi.org/10.15537/smj.2017.11.20760 | No eligible population: primary healthcare centers |
| 17 | Nouwens, E., van Lieshout, J., Bouma, M., Braspenning, J., & Wensing, M. (2014). Effectiveness of improvement plans in primary care practice accreditation: a clustered randomized trial. PloS one, 9(12), e114045. https://doi.org/10.1371/journal.pone.0114045 | No eligible population: primary care practice accreditation |
| 18 | AlRubaey et al 2018 The Impact of Accreditation of Primary Healthcare Centers on Quality of Care as Perceived by Healthcare Providers and Directors in Baghdad. IRAQI JOURNALOF COMMUNITY MEDICINE, 31(1), 1-5 | No eligible population: Primary Health Care centers |
| 19 | Wagner et al (2012) Joint Commission Accreditation and Quality Measures in U.S. Nursing Homes. Policy, Politics, & Nursing Practice, 13(1) 8–16. | No eligible population: Nursing Homes accreditation |
| 20 | Wagner (2012) Impact of Voluntary Accreditation on Deficiency Citations in U.S. Nursing Homes. The Gerontologist, 52(4), 561–570. | No eligible population: Nursing home accreditation |
| 21 | Saleh et al 2014 Beyond Accreditation A Multi-Track Quality-Enhancing Strategy for Primary Health Care in Low-and Middle-Income Countries. International Journal of Health Services, 44(2), 355–372 | No eligible population: Primary Health Care setting |
| 22 | Tabrizi, J. S., Gharibi, F., & Pirahary, S. (2013). Developing of National Accreditation Model for Rural Health Centers in Iran Health System. Iranian journal of public health, 42(12), 1438–1445. | No eligible population:  Rural primary Health Care centers |
| 23 | Hemadeh et al 2019 Patient satisfaction with primary healthcare services in Lebanon. International Journal of Health Planning and Management;34, e423–e435 | No eligible population: Primary Health Care services |
| **No eligible intervention: (n = 46)** | | |
| 1 | Murphy et al. (2013) Three Decades of Quality Improvement in Electroconvulsive Therapy: Exploring the Role of Accreditation The Journal of ECT. 29(4):312–317,  doi: 10.1097/YCT.0b013e3182926fc4 | No eligible intervention: single-specialty accreditation programmes Electroconvulsive Therapy Accreditation Service (ECTAS) |
| 2 | Chaplin et al 2018 The Impact of Accreditation for 10 Years on Inpatient units for adults of working age in the UK. Psychiatric Services; 69:1053–1055; doi: 10.1176/appi.ps.201700567 | No eligible intervention:  single-specialty (Psychiatric Services accreditation program) |
| 3 | Alcazar et al. ( 2011) Professional involvement in the design of accreditation manuals. International  Journal of Healthcare Quality Assurance, 24,611-620. | No eligible intervention: design of accreditation manuals |
| 4 | Russell GK, Jimenez S, Martin L, Stanley R, Peake MD, Woolhouse I. A multicentre randomised controlled trial of reciprocal lung cancer peer review and supported quality improvement: results from the improving lung cancer outcomes project. British Journal of Cancer 2014;110(8):1936-42. [DOI: 10.1038/bjc.2014.146] | No eligible intervention: single-specialty accreditation programmes; Surgical Care and Outcomes Assessment Program |
| 5 | Telem DA, Talamini M, Altieri M, et al. The effect of national hospital accreditation in bariatric surgery on perioperative outcomes and long-term mortality. Surg Obes Relat Dis. 2015; 11(4): 749- 757. PMid: 26001555. https://doi.org/10.1016/j.soard.20  14.05.012 | No eligible intervention: single-specialty accreditation programmes |
| 6 | Kwon et al 2013 The Impact of Accreditation on Safety and Cost of Bariatric Surgery. Surgical Obesity Related Disease. 9(5): 617–622 | No eligible intervention:  single-specialty (Surgical Care) accreditation program |
| 7 | Hogden, A, Greenfield, D, Brandon, M, Debono, D, Mumford, V, Westbrook, J & Braithwaite, J 2017, 'How does accreditation influence staff perceptions of quality in residential aged care?', Quality in Ageing and Older Adults, vol. 18, no. 2, pp. 131-144. https://doi.org/10.1108/QAOA-07-2016-0028 | No eligible intervention:  aged care accreditation programme. |
| 8 | Wright, J. R. (2017). The American College of Surgeons, minimum standards for  hospitals, and the provision of high-Quality laboratory services. Archives of Pathology  & Laboratory Medicine, 141(5), 704-717. | No eligible intervention: laboratory services accreditation |
| 9 | Kim YS, Jung SE, Choi BG, Shin YR, Hwang SS, Ku YM, Lim YS, Lee JM. (2010) Image Quality Improvement after Implementation of a CT Accreditation Program. Korean Journal of Radiology. 11(5):553-559. https://doi.org/10.3348/kjr.2010.11.5.553 | No eligible intervention: Radiology accreditation program |
| 10 | Albert & Das 2011 Quality Assessment in Oncology. International Journal Radiation Oncology Biology Physics, Vol. 83, No. 3, pp. 773e781, 2012 | No eligible intervention: quality assessment of oncology accreditation program |
| 11 | Ramadan et al 2011 Quality Assurance of Egyptian Higher Education A Policy Transfer. Literacy Information and Computer Education Journal (LICEJ), 2(1), 338-349 | No eligible intervention:  Higher education accreditation program |
| 12 | Brodribb et al 2013 Baby-Friendly Hospital Accreditation In-Hospital Care Practices and Breastfeeding. PEDIATRICS; 131(4), 685-692 | No eligible intervention:  single-specialty (BFHI) accreditation program |
| 13 | Gharibi, F. & Tabrizi, J. 2018 Development of an accreditation model for health education and promotion programs in the Iranian primary healthcare system a Delphi study. Health Promotion Perspectives, 2018, 8(2), 155-162  doi: 10.15171/hpp.2018.20 | No eligible intervention:  single-specialty Health education promotion accreditation program |
| 14 | Greenfield, D. et al. (2014) A mechanism for revising accreditation standards: a study of the process, resources required and evaluation outcomes. BMC Health Services Research. [Online] 14 (1), 571. [online]. Available from: http://search.proquest.com/docview/1627796772/. | No eligible intervention:  Development of accreditation program |
| 15 | Tack, V., Schuuring, E., Keppens, C., Hart, N. '., Pauwels, P., van Krieken, H., & Dequeker, E. (2018). Accreditation, setting and experience as indicators to assure quality in oncology biomarker testing laboratories. British journal of cancer, 119(5), 605–614. https://doi.org/10.1038/s41416-018-0204-9 | No eligible intervention:  Labaratory accreditation program |
| 16 | Jarvinen H., Wilcox P. (2014) Clinical Audit and Practice Accreditation. In: Lau L., Ng KH. (eds) Radiological Safety and Quality. pp 291-306. Springer, Dordrecht | No eligible intervention: Radiation oncology accreditation |
| 17 | Brinza, E. K., Zhu, L. J., Lilly, M., Manning, W. J., Needleman, L., & Gornik, H. L. (2016). Accreditation is Perceived to Improve the Quality of Vascular Testing Facilities. Journal for Vascular Ultrasound, 40(2), 63–69. https://doi.org/10.1177/154431671604000201 | No eligible intervention: single-specialty (vascular laboratories) accreditation program |
| 18 | Gurdemir, B. and Arıbal, E (2012) Assessment of mammography quality in İstanbul. Diagnostic Interventional Radiology, 18,468–472 | No eligible intervention: Radiology (ACR) accreditation phantom |
| 19 | Maritza et al 2014 Using the ACR CT accreditation phantom for routine image quality assurance on both CT and CBCT imaging systems in a radiotherapy environment. Journal of Applied Clinical Medical Physics, 15(4), 226-239 | No eligible intervention: Radiology (ACR) CT accreditation phantom |
| 20 | Driscoll B., Keller, H., Jaffray, D. & Coolens C. (2013) Development of a dynamic quality assurance testing protocol for multisite clinical trial DCE‐CT accreditation. Medical Physics, 40 (8) | No eligible intervention: Radiology (ACR) CT accreditation phantom |
| 21 | Kim, H ; Malatesta, TM ; Dicker, A ; Simone, Nl ; Den, Rb ; Bar Ad, V (2015) Improving the quality of patient care through a collaborative maintenance of certification and next accreditation system project. Journal of clinical oncology, 33(15). DOI: 10.1200/jco.2015.33.15_suppl.e17728 | No eligible intervention: Accreditation of Graduate Medical Education |
| 22 | Abujudeh et al 2010 Quality Initiatives Key Performance Indicators for Measuring and Improving Radiology Department Performance. RadioGraphics, 30, 571–583 | No eligible intervention: Radiology accreditation program |
| 23 | Qutishat (2009) Medical laboratory quality and accreditation in Jordan. Clinical Biochemistry 42, 256–258 | No eligible intervention: Medical laboratory accreditation program |
| 24 | Birkmeyer et al. (2010) Hospital Complication Rates with Bariatric Surgery in Michigan. Jama-Journal of The American Medical Association, 304(4) 435–442 | No eligible intervention: Bariatric center of excellence accreditation program |
| 25 | Morton, M., M. Garg, and M. Nguyen (2014) Does Hospital Accreditation Impact Bariatric Surgery Safety? Annals of Surgery, 260(3), 504–50 | No eligible intervention: Bariatric center of excellence accreditation program |
| 26 | Bhangu A., Bowley DM, Horner R, Baranowski E, Raman S & Karandikar S. (2012) Volume and accreditation, but not specialty, affect quality standards in colonoscopy. British Journal of Surger, 99(10), 1436-44. doi: 10.1002/bjs.8866. | No eligible intervention:  Healthcare professional accreditation program |
| 27 | Albanese et al. (2014) Baby Heart Project: The Italian Project for Accreditation and Quality Management in Pediatric Cardiology and Cardiac Surgery. Pediatric Cardiology, 35(7), 1162–1173  DOI 10.1007/s00246-014-0910-x | No eligible intervention:  Pediatric Cardiology and Cardiac Surgery accreditation program |
| 28 | Press et al (2011) The Impact of Resident Duty Hour Reform on Hospital Readmission Rates Among Medicare Beneficiaries. Journal of General Internal Medicine 26(4), 405–11 | No eligible intervention: Graduate Medical Education accreditation program |
| 29 | Takenori et al (2018) The Impact of Joint Commission International Accreditation on Time Periods in the Operating Room: A Retrospective Observational Study. PLoS ONE, 13(9) e0204301 | No eligible intervention: Surgical accreditation program (one location CBA study design) |
| 30 | Park et al (2015) Evaluation of the Image Quality in Digital Breast Tomosynthesis (DBT) Employed with a Compressed-Sensing (CS)-Based Reconstruction Algorithm by Using the Mammographic Accreditation Phantom. Nuclear Inst. and Methods in Physics Research, A (804), 72–78 | No eligible intervention: Mammographic accreditation program |
| 31 | Al Khalifah et al (2019) Validity of Using Accreditation Phantom in Quality Control of Digital Tomosynthesis. Journal of Allied Health 48.1 E15–E19 | No eligible intervention: Mammographic accreditation program |
| 32 | Frank et al (2012) Accreditation and quality assurance for professional degree programmes: comparing approaches in three European countries, Quality in Higher Education, 18(1), 75-95, DOI: 10.1080/13538322.2012.669910 | No eligible intervention:  accreditation for professional degrees |
| 33 | Whitehead et al 2014 Compassionate Care? A Critical Discourse Analysis of Accreditation Standards. Medical education. 48(6), 632–643. | No eligible intervention: professional training accreditation program |
| 34 | Sharma and Fletcher 2014 A review of echocardiography in anaesthetic and peri-operativepractice. Part 2: training and accreditation | No eligible intervention: Echocardiography accreditation program |
| 35 | Merkow et al 2014 Relationship Between Cancer Center Accreditation and performance on publicaly reported quality measure. Annals of Surgery, 259(6) | No eligible intervention:  Cancer Center Accreditation |
| 36 | Huang et al (2016) Maternity Staff Perspectives Regarding Resource Demands of Breastfeeding Supportive Practices in Accordance with the Baby-Friendly Hospital Initiative Accreditation: a Q Methodology Approach. Journal of advanced nursing, 72(6), 1301–1312 | No eligible intervention:  Baby-Friendly Hospital Accreditation |
| 37 | Yao et al 2010 Improving Quality Management Systems of Laboratories in Developing Countries An Innovative Training Approach to Accelerate Laboratory Accreditation. American Journal of Clinical Pathology, 134, 401-409 | No eligible intervention:  Hospital Laboratory Accreditation |
| 38 | Yanikkaya-Demirel 2009 ISO 15189 accreditation Requirements for quality and competence of medical laboratories, experience of a laboratory II. Clinical Biochemistry 42, 279–283 | No eligible intervention:  Hospital Laboratory Accreditation |
| 39 | Berwouts et al 2010 Approaches to quality management and accreditation in a genetic testing laboratory. European Journal of Human Genetics, 18, S1–S19 | No eligible intervention: accreditation of medical laboratories |
| 40 | Peter et al 2010 Impact of Laboratory Accreditation on Patient Care and the Health System. American Journal of Clinical Pathology, 134, 550-555 | No eligible intervention: accreditation of medical laboratories |
| 41 | Sisay, A., Mindaye, T., Tesfaye, A., Abera, E., & Desale, A. (2015). Assessing the outcome of Strengthening Laboratory Management Towards Accreditation (SLMTA) on laboratory quality management system in city government of Addis Ababa, Ethiopia. The Pan African medical journal, 20, 314. https://doi.org/10.11604/pamj.2015.20.314.5375 | No eligible intervention: accreditation of medical laboratories |
| 42 | Gilliland et al 2016 Development and Implementation of a Quality Improvement Process for Echocardiographic Laboratory Accreditation. Journal of the American Society of Echocardiography, 33, (3), 459-471 | No eligible intervention:  Laboratory Accreditation |
| 43 | Wang, H. F., Jin, J. F., Feng, X. Q., Huang, X., Zhu, L. L., Zhao, X. Y., & Zhou, Q. (2015). Quality improvements in decreasing medication administration errors made by nursing staff in an academic medical center hospital: a trend analysis during the journey to Joint Commission International accreditation and in the post-accreditation era. Therapeutics and clinical risk management, 11, 393–406. https://doi.org/10.2147/TCRM.S79238 | No eligible intervention:  medication administration accreditation program |
| 44 | Alomi et al 2018 National Survey of Pharmacy Practice at MOH Hospitals in Saudi Arabia 2016-2017: Clinical Pharmacy Services. Journal of Pharmacy Practice and Community Medicine. 4(1s):S01-S08 | No eligible intervention:  Pharmacy Practice accreditation program |
| 45 | Nguyen et al 2012 Outcomes of Bariatric Surgery Performed at Accredited vs Nonaccredited Centers. Journal of American College of Surgeons, 215(4), 467–474 | No eligible intervention: Bariatric surgery centers accreditation |
| 46 | Woodcock et al 2010 The role of standards and training in preparing for accreditation. American Journal of Clinical Pathology;134, 388-392  388 DOI: 10.1309/AJCP03TFPBKEYYNT | No eligible intervention:  Laboratory accreditation program |
| **Inadequate outcome: (n=43)** | | |
| 1 | Hinchcliff et al (2012) Evaluation of current Australian health service accreditation processes  (ACCREDIT-CAP): protocol for a mixed-method research project | Inadequate outcome: a study protocol |
| 2 | Correa et al. (2018) The Influence of Accreditation on the Sustainability of Organizations with the Brazilian Accreditation Methodology. Journal of Healthcare Engineering, https://doi.org/10.1155/2018/1393585 | Inadequate outcome:  Neither about accreditation impact nor identified influencing factors of accreditation implementation |
| 3 | Andersen MK, Pedersen LB, Siersma V, et al. Accreditation in general practice in Denmark: study protocol for a cluster randomized  controlled trial. Trials. 2017;18(1):69. DOI 10.1186/s13063-017-1818-6 | Inadequate outcome:  a study protocol |
| 4 | Greenfield D, Pawsey M, Naylor J, Braithwaite J. Are accreditation surveys reliable? Int J Health Care Qual Assur. 2009;22(2):105‐116. | Inadequate outcome:  not relevant to the review objectives |
| 5 | Braithwaite, J., Greenfield, D., Westbrook, J., Pawsey, M., Westbrook, M., Gibberd, R.,  Naylor, J., Nathan, S., Robinson, M., Runciman, B., Jackson, M., Travaglia, J., Johnston, B., Yen, D., McDonald, H., Low, L., Redman, S., Johnson, B., Corbett, A., Hennessy, D., Clark, J., Lancaster, J. (2010). Health service accreditation as a predictor of clinical and organisational performance: a blinded, random, stratified study. Quality and Safety Health Care, 19(1), 14-21. | Inadequate outcome:  examined validity of accreditation as a predictor of healthcare performance |
| 6 | Elkins G, Cook T, Dove J, et al. Perceived stress among nursing and administration staff related to accreditation. Clin Nurs Res. 2010;19(4):376‐386. | Inadequate outcome:  not relevant to the review objectives |
| 7 | Freire, E., Batista, R., & Martinez, M. (2016). Project management for hospital accreditation: a case study. Online Brazilian Journal of Nursing, 15(1), 96-108. doi:https://doi.org/10.17665/1676-4285.20165158 | Inadequate outcome:  not relevant to the review objectives |
| 8 | De oliveira JLC, Gabriel CS, Fertonani HP, Matsuda LM. (2017) Management changes resulting from hospital accreditation. Revista Latino-Americana de Enfermagem.;25:e2851. | Inadequate outcome:  not relevant to the review objectives |
| 9 | Gabriel et al 2017 Brazilian Nurses' Perspective on the Impact of Hospital Accreditation | Inadequate outcome: not relevant to the review objectives |
| 10 | Diab, S.M. (2011), “The extent to which Jordanian doctors and nurses perceive the accreditation in  private hospitals”, International Journal of Marketing Studies, Vol. 3 No. 1, pp. 78-94. | Inadequate outcome:  Discuss health professionals perception about the  accreditation |
| 11 | Greenfield, D., Pawsey, M., Hinchcliff, R., Moldovan, M. and Braithwaite, J. (2012), The standard of healthcare accreditation standards: a review of empirical research underpinning their development and impact | Inadequate outcome:  Examined the development methods and application of healthcare accreditation standards |
| 12 | Jaafaripooyan E. (2014) Potential pros and cons of external healthcare performance  evaluation systems: real-life perspectives on Iranian  hospital evaluation and accreditation program | Inadequate outcome:  Identified the potential pros and cons of Accreditation |
| 13 | Jaafaripooyan et al 2011 Healthcare accreditation systems further perspectives on performance measures | Inadequate outcome:  Identified a number of performance measures to facilitate the evaluation of accreditation programs. |
| 14 | Touati, N. and Pomey, M. (2009), “Accreditation at a crossroads: are we on the right track?”, Health Policy, Vol. 90 No. 2, pp. 156-165. | Inadequate outcome:  Evaluates the extent to which the accreditation process acts as a tool for bureaucratic coercion as opposed to a tool for learning. |
| 15 | Guérin S, Le Pogam M-A, Robillard B, et al. Can we simplify the hospital accreditation process? Predicting accreditation decisions from a reduced dataset of focus priority standards and quality indicators: results of predictive modelling. BMJ Open. 2013;3:e003289. | Inadequate outcome:  not relevant to the review objectives |
| 16 | Devkaran et al 2019 Impact of repeated hospital accreditation surveys on quality and reliability, an 8-year interrupted time series analysis. BMJ Open; 9, e024514. doi:10.1136/ bmjopen-2018-024514 | Inadequate outcome:  not relevant to the review objectives  it is evaluating the validity  of the four phases of the re-accreditation life cycle program not the effect of accreditation |
| 17 | Braithwaite, J., Shaw, C., Moldovan, M., Greenfield, D., Hinchcliff, R., Mumford, V., Kristensen, MB., Westbrook, J., Nicklin, W., Fortune, T., Whittaker, S. (2012). Comparison of health service accreditation programmes in low and middle income countries with those in higher income countries: a cross-sectional study. International Journal for Quality in Healthcare, 24(6), 568-577. | Inadequate outcome:  not relevant to the review objectives |
| 18 | Camillo, N. R. S., Oliveira, J. L. C. D., Bellucci Junior, J. A., Cervilheri, A. H., Haddad, M. D. C. F. L., Matsuda, L. M. (2016). Accreditation in a public hospital: perceptions of a multidisciplinary team. Revista brasileira de enfermagem, 69(3), 451-459. | Inadequate outcome: discussed accreditation in general and identified advantages offered by the Accreditation |
| 19 | Haj-Ali et al 2014 Exploring the relationship between accreditation and patient satisfaction the case of selected Lebanese hospitals. Int J Health Policy Manag., 3(6), 341–346 doi 10.15171/ijhpm.2014.116 | Inadequate outcome:  Assessed the relationship  between patient satisfaction and accreditation without reporting enough data about the review objectives |
| 20 | De Oliveira, J., de Magalhães, A., Bernardes, A., Haddad, M., Wolff, L., Marcon, S. S., & Matsuda, L. M. (2019). Influence of hospital Accreditation on professional satisfaction of the nursing team: mixed method study. Revista Latino-Americana de Enfermagem, 27, e3109. https://doi.org/10.1590/1518-8345.2799.3109 | Inadequate outcome:  not relevant to the review objectives |
| 21 | Tabrizi, J. S., Gharibi, F., & Wilson, A. J. (2011). Advantages and disadvantages of health care accreditation models. Health Promotion Perspectives, 1(1), 1. | Inadequate outcome: discussed accreditation in general and identified advantages and disadvantages of different accreditation models |
| 22 | Schmaltz, S.P., Williams, S.C., Chassin, M.R., Loeb, J.M. and Wachter, R.M. (2011) Hospital performance trends on national quality measures and the association with joint commission accreditation. Journal of Hospital Medicine, 6(8), 454-461. | Inadequate outcome: Compared the difference in development of accredited vs. non accredited hospitals, not the effects of accreditation |
| 23 | Shaw et al 2010 Sustainable healthcare accreditation messages from Europe in 2009. International Journal for Quality in Health Care 2010; 22 (5), 341 –350 | Inadequate outcome: Described the development of national accreditation organizations in Europe to identify trends over time using data from previous surveys. |
| 24 | Teymourzadeh et al 2017 Surveyor Management of Hospital Accreditation Program A Thematic analysis conducted in Iran. Iran Red Crescent Medical Journal; 18(5): e30309. doi: 10.5812/ircmj.30309 | Inadequate outcome: Identified the dimensions and factors affecting surveyor management of hospital accreditation programs |
| 25 | Oliveirs, J. and Matsuda, L. 2016 Disqualification of certification by hospital accreditation: Perceptions of professional staff. Texto & Contexto - Enfermagem, 25(1), e4430014. Epub April 01, 2016.https://doi.org/10.1590/0104-07072016004430014 | Inadequate outcome:  not relevant to the review objectives |
| 26 | Chuang, S., Howley, P. and Gonzales, S. 2019 An international systems-theoretic comparison of hospital accreditation: developing an implementation typology, International Journal for Quality in Health Care; 31(5), 371–377, https://doi.org/10.1093/intqhc/mzy189 | Inadequate outcome:  not relevant to the review objectives |
| 27 | ANDRES, E.B., SONG, W., SONG, W. and JOHNSTON, J.M., 2019. Can hospital accreditation enhance patient experience? Longitudinal evidence from a Hong Kong hospital patient experience survey. BMC health services research, 19(1), pp. 623. | Inadequate outcome:  not relevant to the review objectives |
| 28 | Auras S, Geraedts M. Patient experience data in practice accreditation—an international comparison. Int J Qual Health Care. 2010;22(2):132-139. | Inadequate outcome:  not relevant to the review objectives |
| 29 | Petrusevska A, Dimitrievska V, Zisovska E. & Spasovski M. (2016) Standpoint of medical staff about accreditation in a pre-accreditation period in hospitals in Republic of Macedonia. Int J Sci Basic Appl Res.;26(2):242-251. | Inadequate outcome:  not relevant to the review objectives |
| 30 | Falstie-Jensen et al (2015) Compliance with hospital accreditation and patient mortality: a Danish nationwide population-based study. International Journal for Quality in Health Care, 27(3), 165–174 https://doi.org/10.1093/intqhc/mzv023 | Inadequate outcome: |
| 31 | Flotta et al (2012) Appraising Hospital Performance by Using the JCHAO/CMS Quality Measures in Southern Italy. Quality of Care in Italy journal, 7(11), e48923 | Inadequate outcome:  not relevant to the review objectives |
| 32 | Mohebbifar et al 2017 Association between Hospital Accreditation and Patient Satisfaction: A Survey in the Western Province of Iran. Bangladesh Journal of Medical Science. 16 (1 ), 77-84 | Inadequate outcome:  The main focus is on patient satisfaction, no enough data reported on accreditation. |
| 33 | Bogh, S. B., Falstie-Jensen, A. M., Bartels, P., Hollnagel, E. and Johnsen, S. P. (2015), ‘Accreditation and improvement in process quality of care: a nationwide study’, International Journal for Quality in Health Care. 27(4), 336-43. | Inadequate outcome:  not relevant to the review objectives  Compared the difference in performance measures improvement in accredited vs. non accredited hospitals, not the effects of accreditation |
| 34 | Greenfield D, Pawsey M, Braithwaite J. (2011) The role and impact of accreditation on the healthcare revolution. . Revista acreditação, 1(2), 134-150 | Inadequate outcome:  not relevant to the review objectives |
| 35 | Hayati IN, Azimatun N, Rozita H, Ezat W, Rizal A. In‐patient's satisfaction in the medical and surgical wards—a comparison between accredited and non-accredited hospital in the state of Selangor. Journal Kesihatan Masyarakat. 2010; 16(1):60‐68. | Inadequate outcome:  The main focus is on patient satisfaction, no enough data reported on accreditation. |
| 36 | Bogh et al 2018 Hospital accreditation Staffs experience and perceptions. International Journal of Health Care Quality Assurance, 31(4) DOI: 10.1108/IJHCQA-06-2017-0115 | Inadequate outcome:  Focus on Staffs understanding of hospital accreditation in general |
| 37 | Bogh, S. B., Falstie-Jensen, A. M., Hollnagel, E., Holst, R., Braithwaite, J., Raben, D. C. and Johnsen, S. P. (2017), ‘Predictors of the effectiveness of accreditation on hospital performance: A nationwide stepped wedge , International Journal for Quality in Health Care, pp. 1-7 | Inadequate outcome:  not relevant to the review objectives |
| 38 | Touati N, Pomey M-P. Accreditation at a crossroads: are we on the right track? Health Policy 2009; 90:156–65. | Inadequate outcome:  Comparison between Canada and France accreditation programs |
| 39 | Algahtani et al 2017 Perception of hospital accreditation among healthcare professionals in Saudi Arabia. Ann Saudi Med 2017; 37(4): 326-332  DOI: 10.5144/0256-4947.2017.326 | Inadequate outcome:  Explored views of health professionals toward accreditation in general |
| 40 | Sack C, Scherag A, Lutkes P, Günther W, Jöckel KH, Holtmann G. (2011) Is there an association between hospital accreditation and patient satisfaction with hospital care? A survey of 37,000 patients treated by 73 hospitals. Int J Qual Health Care, 23, 278-283. | Inadequate outcome:  Assessed the relationship  between patient satisfaction and accreditation without reporting enough data about the review objectives |
| 41 | Saut et al (2017) Evaluating the impact of accreditation on Brazilian healthcare organizations A quantitative study. International Journal for Quality in Health Care, 29(5), 713–721 | Inadequate outcome:  the study has not used outcome measures to evaluate the accreditation impact |
| 42 | Shaw, C., Groene, O., Mora, N. and Sunol, R. (2010), “Accreditation and ISO certification: do they explain differences in quality management in European hospitals?”, International Journal for Quality in Health Care, Vol. 22 No. 6, pp. 445-451. | Inadequate outcome:  the study compares between Accreditation and ISO certification programs  in hospitals |
| 43 | Shaw CD, Groene O, Botje D, et al. (2014) The effect of certification and accreditation on quality management in 4 clinical services in 73 European hospitals. Int J Qual Health Care.; 26: 100-107. PMid: 24615598. https://doi.org/10.1093/intqhc/mzu023 | Inadequate outcome:  No data reported on effect of certification and accreditation with clinical outcomes |
| **Letter/opinion/editorial/ conference reports/** **Commentary/** **abstract format (complete study not available) (n = 14)** | | |
| 1 | Greenfield D, Braithwaite J. Developing the evidence base for accreditation of healthcare organisations: a call for transparency and innovation. Qual Saf Health Care 2009; 18:162–3. | Editorial report |
| 2 | Garvey, C, J., Cook, J.V., Wiltsher, C. & Whitley, S. (2009) Radiology accreditation—towards a safer quality service. Clinical Radiology Journal; 64(9), 853-856. https://doi.org/10.1016/j.crad.2009.04.003 | Editorial report |
| 3 | Bahadori & Hosseini 2017 Whether Hospital Accreditation Has Led to Improvement of Care Quality in Iran or not. Hospital Practices and Research; 2(1),1-1 | Editorial report |
| 4 | Aryankhesal 2016 Strategic Faults in Implementation of Hospital Accreditation programs in developing countries Reflection on the Iranian experience. International Journal of Health Policy and Management; 5(9), 515–517 | Editorial report |
| 5 | Poku et al 2017 Hospital Accreditation and Community Health. The American Journal of Medicine; 130(2) | Commentary report |
| 6 | Anon (2010) Joint Commission to accredit medical homes. (HEALTH POLICY)(Brief article). 87 (21), 21. | Letter (Brief article) |
| 7 | Wood 2009 Competency-based Training Accreditation as a Pathway to Wisdom. Radiology; 252,322–323 | Commentary paper |
| 8 | Garvey et al (2009) Radiology accreditation towards a safer quality service. Clinical Radiology 64, 853e856 | Editorial report |
| 9 | Wen, C., Iqbal, U., & Li, Y. (2017) Healthcare Quality Improvements through Hospital Accreditation Compliance and Effective Procedure Use. International Journal for Quality in Health Care 29(5) 603–603. | Editorial report |
| 10 | Jha (2018) Accreditation, Quality, and Making Hospital Care Better. JAMA, 320(23) 2410–2411 | Opinion Piece |
| 11 | Ott 2016 What Can You Do, Follow Written Protocols. Journal of Radiology Nursing, 35, 321e322 | Letter/ opinion |
| 12 | Wong et al (2015) Quality Assurance Peer Review Chart Rounds: Resident and Faculty Experiences in Relation to the (ACGME) Accreditation Council for Graduate Medical Education Competencies. International Journal of Radiation Oncology, Biology, Physics 93.3: E373–E374 | Abstract format |
| 13 | Smits et al 2014 Hospital accreditation lessons from low- and middle-income countries. Smits et al. Globalization and Health Journal, 10, 65 | Commentary paper |
| 14 | Mate, K. S., Rooney, A. L., Supachutikul, A., Gyani, G. (2014). Accreditation as a path to achieving universal quality health coverage. Globalization and Health, 10(1), 68. | Commentary paper |
| **Ineligible study design (n = 5)** | | |
| 1 | Petrovic et al 2018 The impact of accreditation on health care quality in hospitals. Vojnosanit Pregl, 75(8), 803–808. | Ineligible study design:  CBAs include one location only in both control and intervention groups |
| 2 | Barghouthi ED, Imam A (2018) Patient Satisfaction: Comparative Study between Joint Commission International Accredited and Nonaccredited Palestinian Hospitals. Health Sci J., 12(1), 547. | Ineligible study design:  CBAs include one location only in both control and intervention groups |
| 3 | Al-Qahtani MF, Al-Dohailan SK, Al-Sharani HT, Al-Medaires MA, Khuridah EN, Al-Dossary NM. (2012) The impact of the status of hospital accreditation on patient satisfaction with the obstetrics and gynecology clinics in the eastern province, Saudi  Arabia. J Med Med Sci., 3(10), 665-673. | Ineligible study design:  Study with only one  intervention or control  site |
| 4 | Al Awa, B., Jacquery, A., Al mazrooa, A., Habib, H., Al Noury, K., El Deek, B., El Hati, T., Devreux, I. (2011). Comparison of patient safety and quality of care indicators between pre and post accreditation periods in King Abdulaziz University hospital. Journal of Medical Sciences, 5(1), 61-66. | Ineligible study design:  Study with only one  intervention or control  site |
| 5 | Al Shammari et al 2015 Impact of Hospital Accreditation on Patient Safety in Hail City Saudi arabia nurse’s perspective. Journal of Nursing and Health Science, 4(1), 51-55 | Ineligible study design:  Study with only one  intervention or control  site |
