# Supplementary material for: Impact of hospital accreditation on quality improvement in healthcare: A systematic review
Source: PLoS One. 2023 Dec 5;18(12):e0294180. doi: 10.1371/journal.pone.0294180 (PMC10697559; doi:10.1371/journal.pone.0294180)
Supplement: S8 File — (DOCX) [file pone.0294180.s008.docx]

**S8: Summary of All Articles Included in the Final Review (n = 21)**

| **No** | **Author(s) / Journal** | **Year** | **Country** | **Setting and participants** | **Study aim(s)** | **Study design** | **Outcomes- factors affecting implementation** | **Quality appraisal results (Maximum score = 36)** |
| --- | --- | --- | --- | --- | --- | --- | --- | --- |
| 1 | Ajarmah & Hashem  European Scientific Journal | 2015 | Jordan | 74 Accredited and non-accredited hospitals  1000 questionnaires were distributed to stratified random sample of hospitals' inpatients. | Compare accredited and non-accredited hospitals in Jordan in terms of patient satisfaction, and investigate the impact of the accreditation programme on patient satisfaction. | A quantitative methodology. descriptive cross-sectional research.  Patient satisfaction was measured using the SERVQUAL scale (tangibility, reliability, responsiveness, assurance and empathy).  T test was used to test the main hypotheses. | There is substantial evidence that accreditation improves patient satisfaction significantly. | 28 |
| 2 | Almasabi & Thomas  International Journal of Health Planning and Management | 2017 | Saudi Arabia | Three accredited public hospitals | Assess the impact of CBAHI on the quality of care. | A mixed method approach involving surveys (n=669), documentary analyses and semi-structure interviews (n=12). | Despite some procedure improvements, CBAHI does not monitor the continuity of health care delivery and had no result on quality outcomes in our analysis. | 33 |
| 3 | Bogh et al.  International Journal for Quality in Health Care | 2016 | Denmark | 25 public hospitals in  Denmark | Examine the changes in hospital care quality over time in relation to the first accreditation cycle of a mandatory accreditation programme. | A multi-level longitudinal stepped-wedge survey design; a type of RCT. | Throughout the study period, the hospital's quality of care improved. When comparing the period during accreditation to the period prior to accreditation, the overall positive change in trend odds ratio (OR = 1.002 per week; 95% confidence interval [CI]: 0.997- 1.006) was not significant.  The authors discovered a significantly reduced trend (OR=0.994 per week; 95% CI: 0.988-0.999) when comparing the post-accreditation period to the period during the accreditation period. As a result, the quality of care continued to improve, albeit at a slower rate than during the accreditation period. | 32 |
| 4 | Davis et al.  American journal of public health | 2009 | USA | State and local health departments health | Determine the most probable incentives to encourage voluntary participation in the national public health accreditation model. | Mixed methods approach  Used both qualitative and quantitative data collection methods (discussion groups and a survey) | As important incentives, there are financial incentives for accredited agencies, financial incentives for agencies considering accreditation, and infrastructure and quality improvement.  Respondents also indicated that grant administration and grant application would encourage their participation in the national accreditation model. Respondents also indicated that technical assistance and training would encourage their participation in the national accreditation model. | 32 |
| 5 | Desveaux et al.  International Journal for Quality in Health Care | 2017 | Canada | HCOs that had participated in Accreditation Canada’s Qmentum program during  2014–2016.  Individuals who had coordinated the accreditation process or were involved in managing or promoting quality. | Investigate how organisations respond to and interact with the accreditation process, as well as the actual and potential mechanisms by which accreditation may influence quality. | Qualitative systematic grounded theory study. | The accreditation process is primarily viewed as a quality assurance process, with feedback frequently feeding into quality improvement activities if it aligns with organisational priorities.  For accreditation to have an impact on quality, three key stages are required: coherence, organisational buy-in, and organisational action. | 27 |
| 6 | Devkaran & O’Farrell  BMC Health Services Research | 2015 | United Arab Emirates | A private multispecialty hospital | Examine the effect of healthcare accreditation on hospital quality measures. | Interrupted time series  analysis - a type of quasi-experimental research  design.  The quality performance outcomes were observed over a 48-month period. | According to the study findings, preparing for the accreditation survey results in significant improvement, with 74% of the measures showing a significant positive pre-accreditation slope.  Accreditation had a greater significant negative effect (48% of measures) than a positive effect (4%) on the performance slope after accreditation. Similarly, after the accreditation survey, there was a larger significant negative change in level (26%) than positive (7%) change.  Furthermore, accreditation had no effect on 11 of the 27 measures. However, three years later, there is still a benefit from accreditation, with performance remaining at around 90%, which is 20% higher than the baseline level in 2009. | 34 |
| 7 | Ehlers et al.  International Journal for Quality in Health Care | 2017 | Denmark | Healthcare professionals/managers in nine selected specialties. | Evaluate hospital employees' attitudes toward accreditation and the Danish Quality Model (DDKM) in Denmark. | A cross-sectional survey. | Overall attitudes were supportive, with physicians more sceptical. Attitudes differed across the five Danish regions and between medical professions. A small group of physicians was extremely negative. | 35 |
| 8 | Halasa et al.  East Mediterranean Health Journal | 2015 | Jordan | 2 private accredited acute general hospitals with matched non-accredited hospitals. | Examine the financial impact of JCI hospital accreditation on five structural and outcome hospital performance measures. | 4-year retrospective comparing study. | Three of the five selected measures demonstrated statistically significant effects (all improvements) associated with accreditation: decreased return to intensive care unit (ICU) within 24 hours of ICU discharge; decreased staff turnover; and completeness of medical records.  Over a three-year period, pooling both hospitals resulted in a total savings of $593 000 in Jordan's health-care system. | 35 |
| 9 | Hijazi et al.  The Journal of Health Care Organization | 2018 | Jordan | Four accredited public hospitals  829 clinical/non-clinical hospital staff members were included in the study. | Examine the impact of applying QM practises on patient centeredness in accredited hospitals, and the differences in the perspectives of various healthcare workers on the attributes affecting patient centred care. | A multiple-case study design with cross-sectional survey. | The importance of the hospital's participation in the accreditation process was shown to be relevant to administrators (gamma = 0.96), nurses (gamma = 0.80), and doctors and other health professionals (gamma = 0.71).  However, administrators (gamma = 0.31) were less likely than nurses (gamma = 0.59), doctors, and other healthcare providers (gamma = 0.55) to perceive the impact of measuring quality improvement outcomes on the delivery of patient-centered care. | 35 |
| 10 | Hinchcliff et al.  BMC Health Services Research | 2013 | Australia | Different healthcare settings.  258 diverse healthcare stakeholders. | Determine the factors that facilitate the effective implementation of accreditation programmes. | A qualitative study.  39 focus groups and eight interviews analyzed thematically | Four critical enablers of effective implementation were identified: the accreditation programme is collaborative, valid, and employs relevant standards; accreditation is well received by health professionals; healthcare organisations are capable of embracing accreditation; and accreditation is appropriately aligned with other regulatory initiatives and supported by relevant incentives. | 36 |
| 11 | Hinchcliff et al.  International Journal for Quality in Health Care | 2016 | Australia | 258 healthcare stakeholders | Investigate how Australian accreditation programmes can promote consumer engagement (CE) in healthcare. | A qualitative study  Forty-seven individual and group interviews | Four mechanisms for CE promotion were discovered. Two of them concerned the need for health-care organisations to meet CE-related standards for consumer experience and satisfaction surveys, as well as consumer participation in organisational governance processes. Two other mechanisms for promoting CE through accreditation processes were identified: consumer participation in standard development and revision, and the implementation of accreditation surveys. | 34 |
| 12 | Lutfiyya et al.  International Journal for Quality in Health Care | 2009 | USA | 730 critical access hospitals | Determine whether the quality measures used in the US Centers for Medicare and Medicaid Services Hospital Compare database differed by JCAHO accreditation status for critical access hospitals. | A cross-sectional survey. | The differences between accredited and non-accredited rural critical access hospitals for 4 out of 16 hospital quality indicators were statistically significant (P ≤ 0.01) and favoured accredited hospitals.  Also, accredited hospitals were more likely to rank in the top half of hospitals for 6 of the 16 quality measures. | 26 |
| 13 | Melo  Journal of Health Organization and Management | 2016 | Portugal | An acute teaching hospital  49 clinical and non-clinical members of staff | Investigate the mechanisms by which accreditation can improve the quality of healthcare services provided. | A qualitative case studies.  46 in-depth semi-structured interviews | According to interviewees, hospital accreditation helped to improve healthcare quality in general, and specifically patient safety, by encouraging staff reflection, higher standardisation of practises, and a greater focus on quality improvement. However, findings indicate that the positive impact of accreditation was due to the approach taken by the hospital in its implementation, as well as the fact that several of the procedures and practises required by accreditation were already in place at the hospital, albeit in an informal manner. | 33 |
| 14 | Mumford et al.  BMJ Open | 2015 | Australia | Six public and private acute care accredited hospitals | Examine the expenses associated with hospital accreditation in Australia. | Mixed methods design;  stakeholder analysis; survey design and implementation; activity-based costs analysis; and expert panel review. | Accreditation costs ranged from 0.03% to 0.60% of total hospital operating costs per year on average over a four-year accreditation cycle.  The survey's years and smaller facilities were associated with higher costs. These costs amount to $A36.83 million on a national scale, or 0.1% of acute public hospital recurrent expenditure in the 2012 fiscal year. | 25 |
| 15 | Pomey et al.  Implementation Science | 2010 | Canada | Five Healthcare  organisations with  different accreditation  statuses. | Examine how the accreditation process aids in the implementation of organisational changes that improve the quality and safety of care. | Retrospective embedded multiple case study  design.  Employed a theoretical framework to analyse various elements and for  each case. | Although accreditation was not always the catalyst for change, it was a highly effective tool for (i) accelerating integration and stimulating a spirit of cooperation in newly merged HCOs; (ii) assisting in the introduction of continuous quality improvement programmes to newly accredited or not-yet-accredited organisations; (iii) creating new leadership for quality improvement initiatives; and (iv) increasing social capital by providing stipends.  The study also discovered that the motivation of HCOs to implement accreditation-related changes waned over time. | 33 |
| 16 | Reisi et al.  International Journal of Health Planning and Management | 2019 | Iran | 43 tertiary public hospitals. | Examine the impact of accreditation on nurses' perceptions of quality of care, and the barriers and facilitators required for effective accreditation implementation. | A cross‐sectional survey | Accreditation benefits had the highest mean score, followed by strategic quality planning, education and training, and staff involvement.  Regression analysis revealed that factors influencing quality results included leadership, commitment, and support; education and training; rewards and recognition; and staff involvement. Financial and capital constraints were encountered. | 34 |
| 17 | Saadati et al.  International Journal of Hospital Research | 2015 | Iran | 14 hospitals | Identify barriers to accreditation promotion in Iranian hospitals and investigate potential solutions. | A qualitative study using a questionnaire  Thematic analysis was used to extract the major concepts. | The most significant challenges to the successful implementation of accreditation were identified as a lack of personnel knowledge, inadequate staff training, and a lack of commitment on the part of managers and physicians. Respondents identified extensive training, participation of chief executives, and adaptation of the hospital's organisational culture as potential drivers of accreditation in Iranian hospitals. | 29 |
| 18 | Saif  International Journal of Business and Management | 2016 | Jordan | Four public hospitals (two accredited hospitals and two non-accredited hospitals).  250 patients | Explore patients' attitudes and beliefs about the quality of hospital services (QHS) and patient satisfaction (PS). | A descriptive-comparative study.  A structured questionnaire was used for data collection. | Patients in both accredited and non-accredited hospitals reported medium levels of QHS and PS; additionally, QHS and PS were significantly correlated (p ≤ 0.05) in both types of hospitals. | 31 |
| 19 | Saleh et al.  International Journal for Quality in Health Care | 2013 | Lebanon | 110 private hospitals | Investigate the perspectives of Lebanese hospitals on the value of accreditation in relation to the associated costs, as well as the type and source of financial investments incurred during the accreditation process. | Mixed methods design; observational cross-sectional design.  The quantitative data analysed using SPSS 19  The qualitative data analysed using thematic analysis | Three-fifths (63% response rate) of responding hospitals saw accreditation as a worthwhile investment. Positive attitudes toward accreditation were primarily related to its impact on improved quality and safety culture. Unfavorable attitudes toward the worthiness of accreditation investment were justified by payers' lack of a link with increased tariffs (25.7%).  Accreditation increased the costs of all hospitals. The highest increases were in staff training (95.7%), consultant costs (80.0%), and infrastructure maintenance (77.1%). Internal absorption (52%) or bank loans (45.7%) covered the majority of hospital expenses. | 28 |
| 20 | Yildiz & Kaya  Clinical Governance: An International  Journal | 2014 | Turkey | JCI accredited university hospital.  258 nurses who started working in the hospital before and continued to work after accreditation | Evaluate nurses' perceptions of the impact of accreditation on quality of care and analyse the impact of accreditation on quality outcomes. | A cross-sectional study.  A questionnaire-based  survey | Nurses were found to have generally high scores for the items pertaining to the benefits of accreditation.  The dependent variable (quality results) and the independent variables had a statistically significant positive correlation (benefits of accreditation and participation of employees). Regression analysis revealed that R2= 0.461 and that the independent variables explained 46.1 percent of the dependent variable, which is a high rate. Patient satisfaction increased following accreditation. | 31 |
| 21 | Zarifraftar  UCT Journal of Management and Accounting Studies | 2018 | Iran | 280 clinical/non clinical staff members from public and private hospital | Ranking the challenges in implementing accreditation standards in private and public hospitals. | A cross-sectional, descriptive research.  A questionnaire  was analysed using various statistical tests | The challenges in implementing accreditation standards were classified into nine aspects, which included encouragement drivers; perception of accreditation standards; financial resources; quality improvement; management knowledge, skills, and commitment; support of regulatory initiatives; standard & surveying development; human resources; and determining macro policies and procedures.  Also discovered were differences in the ranking of these challenges in public and private hospitals. | 32 |
