# Supplementary material for: Impact of hospital accreditation on quality improvement in healthcare: A systematic review
Source: PLoS One. 2023 Dec 5;18(12):e0294180. doi: 10.1371/journal.pone.0294180 (PMC10697559; doi:10.1371/journal.pone.0294180)
Supplement: S9 File — (DOCX) [file pone.0294180.s009.docx]

**S9: Additional references: Included studies in the systematic review but did not cited in the text**

1. Ajarmah BS, Hashem TN. Patient satisfaction evaluation on hospitals; comparison study between accredited and non-accredited hospitals In Jordan. *European Scientific Journal.* 2015;11(32): 298-314.
2. Davis MV, Cannon MM, Corso L, Lenaway D, Baker EL. Incentives to encourage participation in the national public health accreditation model: a systematic investigation. *Am J Public Health*. 2009;99(9):1705-1711. doi:10.2105/AJPH.2008.151118
3. Hinchcliff R, Greenfield D, Hogden A, Sarrami-Foroushani P, Travaglia J, Braithwaite J. Levers for change: an investigation of how accreditation programmes can promote consumer engagement in healthcare. *Int J Qual Health Care*. 2016;28(5):561-565. doi:10.1093/intqhc/mzw074
4. Lutfiyya MN, Sikka A, Mehta S, Lipsky MS. Comparison of US accredited and non-accredited rural critical access hospitals. *Int J Qual Health Care*. 2009;21(2):112-118. doi:10.1093/intqhc/mzp003
5. May CR, Finch T, Ballini L, et al. Evaluating complex interventions and health technologies using normalization process theory: development of a simplified approach and web-enabled toolkit. *BMC Health Serv Res*. 2011; 11:245. Published 2011 Sep 30. doi:10.1186/1472-6963-11-245
6. Mumford V, Greenfield D, Hogden A, et al. Counting the costs of accreditation in acute care: an activity-based costing approach. *BMJ Open*. 2015;5: e008850. doi: 10.1136/bmjopen-2015-008850
7. Pomey MP, Lemieux-Charles L, Champagne F, Angus D, Shabah A, Contandriopoulos AP. Does accreditation stimulate change? A study of the impact of the accreditation process on Canadian healthcare organizations. *Implement Sci*. 2010; 5:31. Published 2010 Apr 26. doi:10.1186/1748-5908-5-31
8. Saadati M, Yarifard K, Azami‐Agdash S, Tabrizi JS. Challenges and potential drivers of accreditation in the Iranian hospitals. *International Journal of Hospital Research.* 2015; 4(1): 37‐42.
9. Saif N. Quality of health services and patients’ satisfaction in accredited and non-accredited hospitals. *International Journal of Business and Management*. 2016; 11(10): 298-305.
