# Supplementary material for: Impact of hospital accreditation on quality improvement in healthcare: A systematic review
Source: PLoS One. 2023 Dec 5;18(12):e0294180. doi: 10.1371/journal.pone.0294180 (PMC10697559; doi:10.1371/journal.pone.0294180)
Supplement: Attachment — Submitted filename: Response to Reviewers.docx [file pone.0294180.s010.docx]

**Response to Reviewers**

**Title:** Impact of hospital accreditation on quality improvement in healthcare: A systematic review

**Version:** 2

**Date:** Oct 08, 2023

**Author's response to reviews:** see over

**Subject:**	Response to reviewer comments for the manuscript “Impact of hospital accreditation on quality improvement in healthcare: A systematic review.”

Dear Academic Editor,

Thank you for the opportunity to revise and resubmit the above manuscript to your esteemed journal. We thank the academic editor and reviewer(s) for their detailed and thorough comments and have addressed their concerns in the revised version of the manuscript. Kindly find below a point-by-point description of the changes made to the manuscript.

**Academic Editor**

**Comment 1:**

1. Please ensure that your manuscript meets PLOS ONE's style requirements, including those for file naming. The PLOS ONE style templates can be found at

<https://journals.plos.org/plosone/s/file?id=wjVg/PLOSOne_formatting_sample_main_body.pdf> and

<https://journals.plos.org/plosone/s/file?id=ba62/PLOSOne_formatting_sample_title_authors_affiliations.pdf>

**Response 1:**

Thank you for highlighting these changes, this has now been completed.

**Comment 2:**

2. Thank you for stating the following in your Competing Interests section:

"NO authors have competing interests"

Please complete your Competing Interests on the online submission form to state any Competing Interests. If you have no competing interests, please state "The authors have declared that no competing interests exist.", as detailed online in our guide for authors at <http://journals.plos.org/plosone/s/submit-now>

This information should be included in your cover letter; we will change the online submission form on your behalf.

**Response 2:**

I appreciate this, I have included this in the cover letter.

**Reviewer comment:**

**Reviewer's Responses to Questions**

**Comments to the Author**

1. Is the manuscript technically sound, and do the data support the conclusions?

The manuscript must describe a technically sound piece of scientific research with data that supports the conclusions. Experiments must have been conducted rigorously, with appropriate controls, replication, and sample sizes. The conclusions must be drawn appropriately based on the data presented.

Reviewer #1: Partly

**Response:**

Many sections within the manuscript have been modified, and more detailed information has been added in order to make it clearer for readers. These changes can be tracked easily in the "Revised Manuscript with Track Changes File.".

2. Has the statistical analysis been performed appropriately and rigorously?

Reviewer #1: I Don't Know

**Response:**

The study is a qualitative systematic review, so it has no statistical analysis.

3. Have the authors made all data underlying the findings in their manuscript fully available?

The PLOS Data policy requires authors to make all data underlying the findings described in their manuscript fully available without restriction, with rare exception (please refer to the Data Availability Statement in the manuscript PDF file). The data should be provided as part of the manuscript or its supporting information, or deposited to a public repository. For example, in addition to summary statistics, the data points behind means, medians and variance measures should be available. If there are restrictions on publicly sharing data—e.g. participant privacy or use of data from a third party—those must be specified.

Reviewer #1: No

**Response:**

Thank you for your comment.

- A summary of the 21 articles included in the final review and the quality assessment results were previously presented in supporting information files (S6 File).
- Per your suggestion, we have uploaded a separate file of included and excluded studies and reasons for exclusion.
- For each of the 21 articles included in the qualitative analysis; review data on the facilitators and barriers to, and the impact of hospital accreditation on quality improvement of healthcare services was extracted, summarized, and coded to the related construct of the NPT framework using a Microsoft Excel Worksheet (**the file uploaded separately**).

4. Is the manuscript presented in an intelligible fashion and written in standard English?

PLOS ONE does not copyedit accepted manuscripts, so the language in submitted articles must be clear, correct, and unambiguous. Any typographical or grammatical errors should be corrected at revision, so please note any specific errors here.

Reviewer #1: Yes

**Response:**

Thank you.

5. Review Comments to the Author

Please use the space provided to explain your answers to the questions above. You may also include additional comments for the author, including concerns about dual publication, research ethics, or publication ethics. (Please upload your review as an attachment if it exceeds 20,000 characters)

**Reviewer #1:**

This is an important paper and review. It is important that there is clarity in the reviewing processes.

**Response:**

Thank you for your kind review. We really appreciate the time and effort which has been taken to review this manuscript.

**Comment 1:**

1. What is the objective for the review as two versions exist?

Objective. To build the evidence for the impact of accreditation on quality improvement of healthcare services and develop an understanding of the contextual factors influencing accreditation implementation in the hospital setting through the lens of Normalisation Process Theory (NPT)

This study provides a comprehensive systematic review to identify contextual factors that may influence hospital accreditation implementation and develop a better understanding of their potential implications

**Response 1:**

Thank you for your comment, the objective was modified in a clearer way throughout the paper; lines 31-34 and lines 113-121.

**Comment 2:**

2. There is no PICOS set out.

**Response 2:**

The PICOS criteria were previously included in table 3 A Complete Descriptions of Inclusion/ Exclusion Criteria for Articles on Accreditation Implementation, however we have made this clearer; we have added a sentence “see table 3 below”; lines 199-201 and written the **PICOS** criteria in **bold** within table 3; page 10.

**Comment 3:**

3) There is no definition of quality improvement or how impact is defined or measured.

**Response 3:**

Per your suggestion we have added a definition of healthcare quality including explanation of its measures; lines 77-87.

**Comment 4:**

4) What setting of healthcare are included or excluded.

**Response 4:**

The included / excluded healthcare settings were previously mentioned in table 3 A Complete Descriptions of Inclusion/ Exclusion Criteria for Articles on Accreditation Implementation;

- Under **Inclusion Criteria** point No. 2. Article conducted in hospital settings (the Population).
- Under **Exclusion Criteria** point No. 3. Article where the population studied was non-hospital setting (e.g., primary healthcare organizations or other community-based healthcare organizations).

, however, we have made this clearer by writing it in bold following your comments; page 10.

**Comment 5:**

5) The reviewing processes - screening and abstraction - how many authors were involved as Cochrane states 2 independent reviewers and extraction in duplicate

**Response 5:**

Thank you for this comment. The section on selection criteria and the data collection process was rewritten to be more explicit, explaining the number of reviewers for both screening eligibility and data extraction from included articles; lines 203-204, lines 209-211, lines 215-216, lines 244-248, and lines 260-261.

**Comment 6:**

6) Why was a qualitative synthesis used when 9 studies used qualitative methods and 4 used mixed methods. How were quantitative data reported?

**Response 6:**

Explaining the reasons behind using qualitative synthesis and the difficulty of conducting a meta-analysis had previously been partially included within the Synthesis of Results section; however, we have made this clearer following your comments; lines 226-235.

For the quantitative studies, the coding and interpretation process of review data focused mainly on the results and discussion parts of each included article. The findings were first coded and brought together in a spreadsheet to better manage the data. Open coding was conducted first, where findings were broken into chunks that relate to different concepts or ideas. Axial coding was then conducted, which involves organizing the emerging concepts into themes. Themes were pre-identified based on the study objectives; lines 244-248 and lines 228-235.

**Comment 7:**

7) SWiM should be used and referenced <https://www.bmj.com/content/368/bmj.l6890>

**Response 7:**

Thank you for your comment. Our systematic review followed the current best practice approach outlined in the Preferred Reporting Items for Systematic Reviews and Meta-Analyses (PRISMA) statement, and the Cochrane Handbook for Systematic Reviews of Interventions (referenced).

In addition, a concerted effort was made to achieve the highest possible degree of objectivity through a combination of reflection; detailed description of the methods used; meticulous implementation of a transparent interpretation process, assessing the veracity of the findings in light of the international work; clear links between the included data, the synthesis, and the conclusions through the theoretical framework of the Normalisation Process Theory, and sufficient reporting of the limitations of the synthesis.

So, we do think we conducted the narrative synthesis with a high degree of clarity and transparency, and we reported this systematic review considering the best practices mentioned above.

**Comment 8:**

8) Data 'were' - sometimes data 'was' is written

**Response 8:**

Thank you for your comment. The use of “was or were” with “data” was reviewed throughout the study and modified accordingly.

**Comment:**

The definitions used are critical to presenting this review and what studies were included or excluded.

**Response:**

Per your suggestion, we have uploaded a separate file of included and excluded studies and reasons for exclusion.

Thank you for providing us with the opportunity to address the reviewers’ comments. We look forward to the outcome of the review process.

Thank you,

Authors
